# Analysis of Paediatric Liaison Service Caseload Trends at King’s College Hospital CAMHS From November 2018 to December 2024: Impact of COVID-19 and Post-Pandemic Recovery

**DOI:** 10.1192/bjo.2025.10500

**Published:** 2025-06-20

**Authors:** Khalid Magzoub, Idura Hisham, Sulagna Chakrabarti

**Affiliations:** South London and Maudsley NHS Trust, London, United Kingdom

## Abstract

**Aims:** To analyse the in-hours emergency/crisis caseload trends of the King’s College Hospital (KCH) CAMHS Paediatric Liaison Service from November 2018 to December 2024, focusing on the impact of the COVID-19 pandemic and the subsequent recovery. The study aims to identify patterns in service demand and contextualise these trends with key pandemic milestones.

**Methods:** Monthly caseload data for in-hours referrals were collected and analysed over a six-year period from November 2018 to December 2024. The data were examined in relation to key events, such as lockdowns, school closures, and reopening phases, to explore potential influences on caseload trends. Median monthly caseloads were calculated, and patterns were compared across different stages of the COVID-19 pandemic and its aftermath.

**Results:** In the pre-COVID phase, monthly caseloads were stable, with a median in-hours emergency referrals of 30. During the COVID phase, caseloads dropped sharply during the first lockdown in March 2020, likely due to school closures and disruptions to referral pathways. Attendance began to recover during the partial reopening of schools in June 2020 but fluctuated with subsequent lockdowns. In the post-COVID recovery phase, caseloads steadily increased but appeared to return to pre-pandemic baselines by 2024. These findings demonstrate a clear relationship between school closures and reduced referrals during COVID lockdowns, as national lockdowns without school closures contributed to higher A&E attendance. This emphasises the critical role of schools in facilitating access to mental health services.

**Conclusion:** This study highlights the significant impact of the COVID-19 pandemic on CAMHS service demand and the resilience of paediatric liaison teams in adapting to fluctuating caseloads. The findings underscore the critical role of schools in identifying and referring young people for mental health support and emphasise the importance of collaborative planning between healthcare and education sectors to prepare for future crises.

